# Strontium Aluminate-Based Long Afterglow PP Composites: Phosphorescence, Thermal, and Mechanical Characteristics

**DOI:** 10.3390/polym13091373

**Published:** 2021-04-22

**Authors:** Anesh Manjaly Poulose, Arfat Anis, Hamid Shaikh, Abdullah Alhamidi, Nadavala Siva Kumar, Ahmed Yagoub Elnour, Saeed M. Al-Zahrani

**Affiliations:** 1 SABIC Polymer Research Center, Department of Chemical Engineering, King Saud University, Riyadh 11421, Saudi Arabia; hamshaikh@ksu.edu.sa (H.S.); AKFHK90@hotmail.com (A.A.); aelnour@ksu.edu.sa (A.Y.E.); szahrani@ksu.edu.sa (S.M.A.-Z.); 2 Department of Chemical Engineering, King Saud University, Riyadh 11421, Saudi Arabia; snadavala@ksu.edu.sa

**Keywords:** long afterglow PP composites, plasticizer, thermal, mechanical

## Abstract

A tremendous potential has been observed in the designing of long afterglow materials for sensing, bioimaging, and encryption applications. In this study, two different strontium aluminate-based luminescent materials; SrAl_2_O_4_: Eu, Dy (S_1_), and Sr_4_Al_14_O_25_: Eu, Dy (S_2_) were melt-mixed with polypropylene (PP) matrix, and the phosphorescence properties were evaluated. After excitation at 320 nm, the PP/S_1_ composite exhibited a green emission and the PP/S_2_ generated a blue emission at 520 nm and 495 nm, respectively. The emission spectra intensity increased by increasing the content of these luminescent fillers. The attenuated total reflection-Fourier transform infrared (ATR-FTIR) experiments show that no chemical reaction occurred during the melt-mixing process. The differential scanning calorimetry (DSC) results revealed that the total crystallinity of the composites reduced by increasing the amount of the fillers; however, no changes in the temperature of melting (Tm) and crystallization (Tc) of PP were observed. Both fillers improved the impact strength of the composites, but the tensile strength (TS) and modulus (TM) decreased. Poly (ethylene glycol) dimethyl ether (P) plasticizer was used to improve the filler-matrix interaction and its dispersion; nevertheless, it adversely affected the intensity of the luminescence emissions.

## 1. Introduction

Luminescent materials emit light, especially in the visible region. When a material continuously emits visible light for longer time (hours) after stopping the radiating source (visible, UV, X-ray, or gamma-ray radiation), a persistence of luminescence or phosphorescence is observed [1]. The first-generation phosphors were Cu or Mn-doped ZnS-based materials (green emission at 530 nm) [2,3]. These materials have been exploited in catalysts and optoelectronic devices. However, their functions are limited because of their low brightness and short afterglow time, and chemical instability in the presence of moisture and CO_2_.

A new luminescence era started with the detection of rare-earth-doped phosphors. The first batch of modern luminescent materials was the rare-earth (R^3+^) and Eu^2+^ doped alkaline-earth aluminates (MAl_2_O_4_:Eu^2+^, R^3+^; M = Ca, Sr, or Ba) [4,5,6,7,8,9]. The Eu^2+^ doped phosphor exhibits a bluish-green luminescence, and the glowing time can be enhanced by adding rare-earth ions, such as neodymium (Nd) or dysprosium (Dy) (e.g., SrAl_x_O_y_: Eu^2+^, Dy^3+^), or by adding Al_2_O_3_ [4,10]. These phosphors have attracted much attention because of their long phosphorescence, greater stability (i.e., moisture and photo-stability), and high quantum efficiency compared with sulfide-based phosphors [9,11,12]. These materials can obtain radiation energy from solar light, remaining photo-luminescent for long periods of time (12–20 h) [13,14]. The luminescent properties of these phosphors allowed their commercial acceptance because of the suitable usage in fluorescent lamps, glowing paints for highways buildings and airports, cathode ray tubes, plasma displays, textile, ceramic area, nighttime clocks, safety displays, among others. Significant growth in the use of these phosphors was observed in optoelectronics, telecommunications, optically active commercial products, and biomedical and way-finding systems [15,16,17].

Recently, various phases of strontium aluminates with rare-earth-doped were developed, such as SrAl_2_O_4_:Eu^2+^, Sr_2_Al_l6_O_11_:Eu^2+^, Sr_4_Al_14_O_25_:Eu^2+^, SrAl_12_O_19_:Ce^3+^, SrAl_12_O_19_:Pr^3+^, and Sr_4_Al_14_O_25_:Sm^2+^ [18,19,20,21]. The wavelength of the emitted visible light is decided by the crystalline phase structure of the resultant strontium aluminate [22,23]. Among the different SrAl_x_O_y_: Eu^2+^, Dy^3+^ phosphors reported, Sr_4_Al_14_O_25_: Eu, Dy, and SrAl_2_O_4_: Eu, Dy have exhibited the strongest potential for long phosphorescence and are commercially available [8,21,24]. Different synthesis techniques such as solid-state reactions [14,20,25], sol-gel method [11,24,26], combustion method [27], solvothermal method [28], chemical precipitation [29], microwave processing, and hydrothermal reaction [30] have been developed. These synthesis methods are often complex and require high temperatures for long-duration phosphorescence materials [31]. A mechanism for luminescence persistence was proposed for SrAl_2_O_4_:Eu^2+^, Dy^3+^ and has been implied to explain the luminescence in several Dy^3+^ and Eu^2+^ co-doped silicates and aluminates. The mechanism is related to the thermally activated release of a hole from Eu^2+^ in its excited 5d state to the valence band, in which it is then trapped by Dy^3+^. Luminescence is generated when the excited electron relaxes back to the ground state of Eu^2+^ [10,32]. The detailed mechanism of the process has been described [15]. However, SrAl_x_O_y_: Eu^2+^, Dy^3+^ phosphors are not exempt from gradual luminescence decay due to their affinity towards moisture. Various encapsulation procedures have been described for gaining stable phosphor, such as Al_2_O_3_ [33], SrF_2_ coating [34], phosphoric acid [35], organic ligands [36], amino-functionalized [37], among others, have been implied and reported. These processes are complicated, require elaborate equipment, and inversely affect the luminescence output. The easy and inexpensive method reported in the literature to prolong the afterglow properties of SrAl_x_O_y_: Eu, Dy is the encapsulation within a polymer matrix, which acts as an insulator for moisture. This process enables the composites to exhibit better chemical stability, good physical properties and can be processed very easily [38,39,40,41,42,43,44,45]. In this study, two different strontium aluminate materials doped with Eu, Dy were incorporated in the selected poly(propylene) matrix, and the phosphorescent characteristics of the resultant composites were studied in detail. Moreover, a known plasticizer poly (ethylene glycol) dimethyl ether (P) was used to enhance the phosphor dispersion in the PP matrix, and also to evaluate its effect on the phosphorescence emission. The characterization studies on these composites provide valuable information on the fabrication of polymer-based luminescent films.

## 2. Materials and Methods

### 2.1. Materials

Poly(propylene) (TASNEE PP H4120) was provided by TASNEE with a density of 0.9 g/cm^3^. It has a melt flow rate (MFR) of 12 g/10 min (ISO 1133). The strontium aluminate phosphors, SrAl_2_O_4_: Eu, Dy (Mw = 209.11 g/mol) (S_1_) and Sr_4_Al_14_O_25_: Eu, Dy (1139.55 g/mol) (S_2_), were supplied by Sigma Aldrich. The plasticizer employed in this study was poly (ethylene glycol) dimethyl ether (P) purchased from Aldrich Company having a number average molecular weight of Mn 1000.

### 2.2. Methods

#### 2.2.1. Preparation of the Composites

Different weight percentages of phosphors (1, 3, 5, and 10) were melt-mixed with the PP matrix in a Polylab QC (Brabender mixer) for a mixing period of 3 min at a temperature of 190–200 °C at 40 rpm. Thin films of 0.5 mm average thickness were made using COLLIN Press, Germany for the phosphorescence measurements.

#### 2.2.2. Characterization of the Composites

Phosphorescence measurements

The phosphorescence tests were carried out in a Fluorescence Spectrophotometer (Agilent Technologies, Santa Clara, CA, USA) using a Xe ultraviolet (UV) lamp. The emission spectra were collected at the wavelength of excitation 320 nm.

Scanning Electron Microscope (SEM)

The morphological and elemental analyses were performed in a JEOL JSM-6360A, Japan SEM model, with energy-dispersive X-ray spectroscopy (EDS) facility. A thin cut surface of the composite was prepared for the analyses. The gold coating for these samples is performed in an auto fine coater (JFC/1600) for 30 s. The coating with gold was carried out to prevent the effect of charging and to improve the quality of the image.

Attenuated Total Reflection-Fourier Transform Infrared Spectroscopy (ATR-FTIR)

ATR-FTIR tests were performed in a Thermo-Scientific Nicolet iN10 FTIR model with germanium micro-tip attachment (400–4000 cm^−1^).

Differential Scanning Calorimetry (DSC)

DSC tests were done in Shimadzu DSC-60A model. Approximately 6–10 mg of sample were taken in an aluminum pan and is heated from 30 °C to 220 °C at a ramp of 10 °C/min with 4 min holding time.

The percent crystallinity was evaluated as follows

$$X_{c}(\%)=\frac{\Delta H_{m}}{(1-\Phi )\Delta H_{m}^{0}}\times 100$$

where (Φ) is the filler weight fraction in the composites, (Δ*H_m_*) is the melting enthalpy, and (Δ*H*^0^_*m*_) is the melting enthalpy of 100% crystalline PP, and was reported as 207 J/g [46].

X-Ray Diffraction (XRD)

The crystalline studies were performed in a wide-angle XRD (Bruker D8 advance). The diffractometer was endowed with a wide-angle goniometer attached to a sealed-tube Cu-Kα radiation source (λ = 1.54056 Å). The scanning was done in the 2θ range of 5° to 50° at 5°/min in the reflection mode.

Thermo-gravimetric analysis (TGA)

TGA was done in a Shimadzu DTG-60H model. For the analysis, 10 ± 1.5 mg of the samples were maintained in an aluminum pan and is heated to a temperature of 600 °C (inert atmosphere) with a heating rate of 20 °C/min; and the loss in weight was monitored.

Mechanical properties

The standard tensile testing specimens (ASTM Type1, Dumb-bell shaped) were prepared using a DSM Xplore, Netherlands (12 cm^3^, microinjection molding). The mold was maintained at room temperature and at a pressure of 6 bar. Tensile testing was performed in Hounsfield H100 KS model UTM (ASTM D638), and the mean of five test results was reported.

## 3. Results

### 3.1. DSC and ATR-FTIR Data

**Figure 1:**
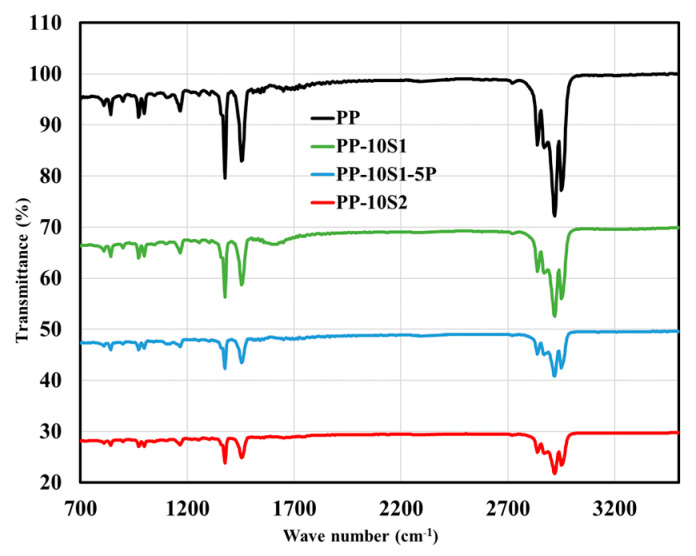
ATR-FTIR spectra of PP, PP/10S_1_, PP/10S_2_, and PP/10S_1_/5P.

The DSC data for the PP and its composites are displayed in Table 1 and Table 2. The presence of both S_1_ and S_2_ did not considerably affect the temperature of melting (T_m_) and crystallization (T_c_) of the composites, demonstrating that the PP was not interacting chemically with S_1_ and S_2_ and the mixing process was purely physical. Additionally, the FTIR spectra also support this observation as PP, PP/10S_1_, PP/10S_2_, and PP/10S_1_/5P have similar FTIR spectra, in which no new peaks were observed nor either peaks diminished (Figure 1). This observation confirms the absence of chemical reactions between both S_1_ and S_2_ and PP. The sharp peaks at 2900 cm^−1^ were due to the asymmetrical CH_2_ bending and the peaks at 1450 cm^−1^ and 1380 cm^−1^ were assigned to the symmetrical CH_3_ bending and asymmetrical CH_3_ bending, respectively, of PP [47].

Conversely, the crystallinity of the composites decreased by increasing the S_1_ and S_2_ contents. The crystallinity decrease was more pronounced in S_2_ because of the bulky chemical structure of S_2_ compared with S_1_, and in turn, restricts the PP chains mobility; thus, decreasing the crystallinity values [48]. The incorporation of the plasticizer in the composites led to a further reduction in the crystallinity percentage of the composites, as shown in Table 1.

### 3.2. X-ray Diffraction Studies of PP, PP/10S_1_, PP/10S_2_, and PP/10S_1_/5P

**Figure 2:**
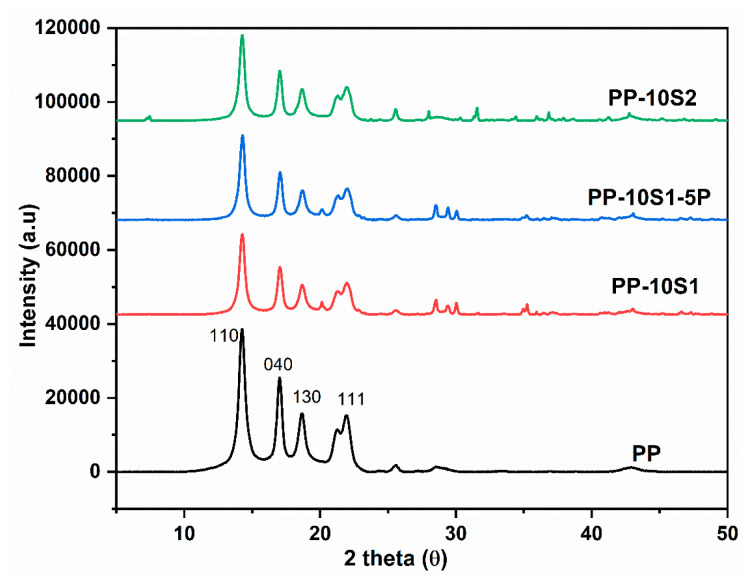
XRD pattern of PP, PP/10S_1_, PP/10S_1_/5P, and PP/10S_2._

Figure 2 illustrates the XRD patterns of neat PP, PP/10S_1_, PP/10S_2_, and PP/10S_1_/5P. All composites show the characteristics diffraction peaks of α-PP, i.e., (110), (040), (130), and (111) [49]. Hence it was clear the absence of chemical reaction between PP and S_1_, S_2_ fillers, or the plasticizer. The (020) peak at 20°; (−211), (220), (211) peaks at 30°, and (031) peaks at 35° are the characteristics diffraction peaks of S_1_ [39], and S_2_ shows characteristics orthorhombic crystal structure with diffraction peaks at 25°, 27°, and 32° [50].

### 3.3. Thermal Gravimetric Analysis (TGA)

**Figure 3:**
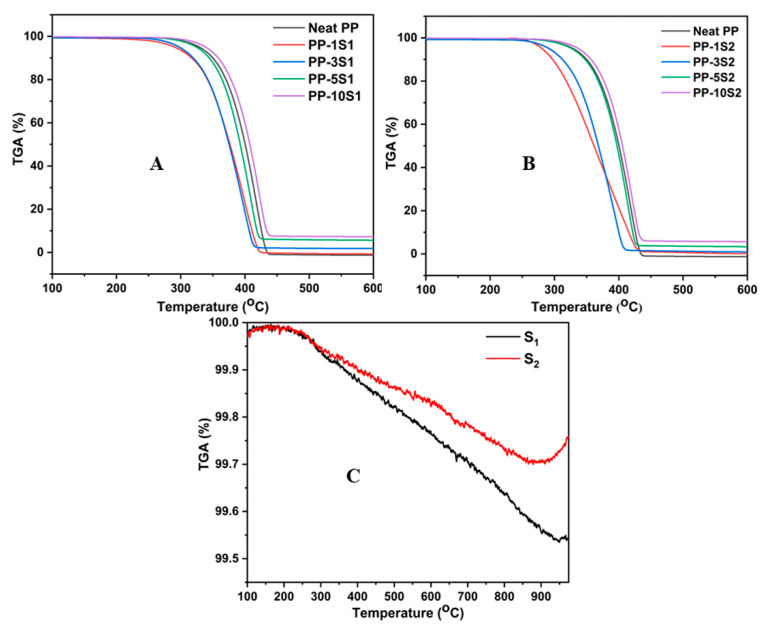
TGA curves of PP/S_1_ (**A**), PP/S_2_ composites (**B**), S_1_ and S_2_ (**C**).

The TGA results collected for PP, PP/S_1_, PP/S_2_, S_1_, and S_2_ under an inert atmosphere are shown in Figure 3A–C, respectively. For both S_1_ and S_2_ composites, the degradation process occurred in a single step and the composite with the highest filler loading have better thermal stability than that of neat PP. The better thermal stability of the composites is because of the fact that the inorganic filler particles (S_1_ and S_2_) can act as a barrier, slowing down the decomposition process of PP [43]. However, in low filler loading concentrations, the thermal stability decreased. Moreover, the residual weight left at the end of the TGA curve was proportional to the loading percentage of the S_1_ and S_2_ fillers. Both S_1_ and S_2_ are inorganic materials and are very much stable as seen from the TGA graph (Figure 3C). The weight loss is very much negligible for both the S_1_ and S_2,_ and on comparing S_1_ and S_2_; S_2_ is found to be slightly more stable than S_1_. The S_1_ and S_2_ are found to be stable until 300 °C and the minor weight loss starts from that temperature, as shown in Figure 3C.

### 3.4. Phosphorescence Emission

**Figure 4:**
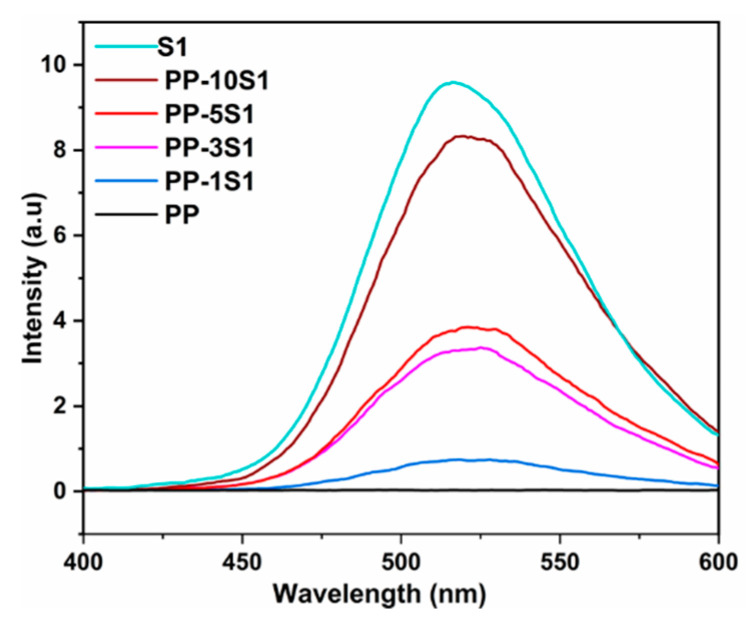
Phosphorescence emissions of S_1_ and PP/S_1_ composites (excitation wavelength: 320 nm; green emission at 520 nm).

**Figure 5:**
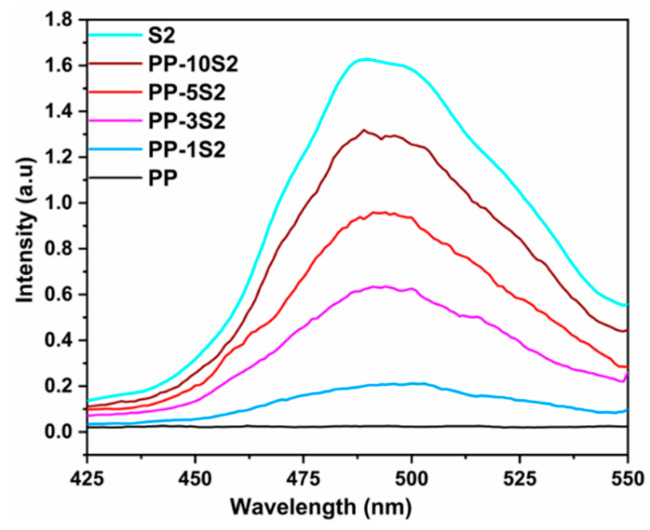
Phosphorescence emissions of S_2_ and PP/S_2_ composites (excitation wavelength: 320 nm; blue emission at 495 nm).

**Figure 6:**
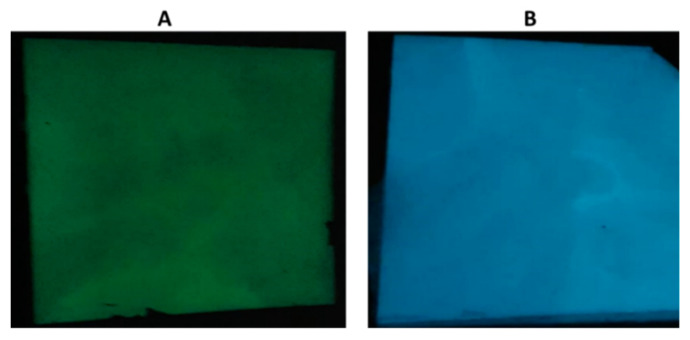
Phosphorescent composites—(**A**) PP/10S_1_ in the dark (green emission) and (**B**) PP/10S_2_ in the dark (blue emission).

The phosphorescence emission spectra of PP/S_1_ and PP/S_2_ composites are presented in Figure 4 and Figure 5, respectively. The emission spectra were collected at an excitation of 320 nm. As expected, the intensity of emission in the spectra of S_1_ and S_2_ were higher than that of PP composites because of the opacity and UV resistance of PP. In the PP/S_1_ and PP/S_2_ composites spectra, the emission intensity increased by increasing the percent loading of both S_1_ and S_2_. The PP/S_1_ composite generated a green emission at 520 nm attributed to the electronic transition of europium divalent ion (Eu^2+^) in the S_1_ phosphors (4f^6^5d^1^ to 4f^7^) [10,51]; the detailed mechanism of phosphorescence has already been described [52]. The PP/S_2_ composites produced a blue emission at 495 nm and the emission intensity increased by increasing the S_2_ content (Figure 5). The green (PP/S_1_) and blue (PP/S_2_) emissions in the dark are shown in Figure 6A,B, respectively.

**Figure 7:**
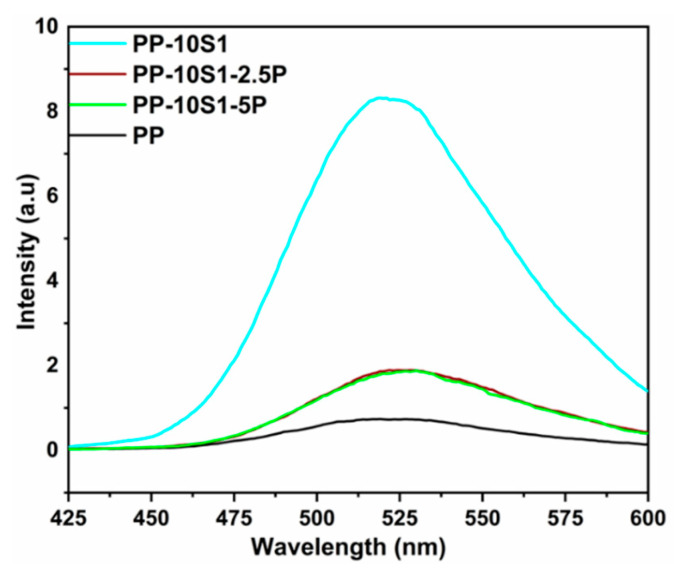
Phosphorescence emission of PP/10S_1_ composites with 2.5 and 5 wt.% P plasticizers (excitation wavelength—320 nm; blue emission at 495 nm).

To investigate the effect of incorporation of plasticizer on the phosphorescence emission, 2.5 and 5 wt.% of plasticizers were added to composite with 10 wt.% of S_1_; however, the plasticizer exhibited an adverse effect on the intensity of emissions. The incorporation of the plasticizer in the 10 wt.% S_1_ composite decreases the overall phosphorescence emission intensity, as shown in Figure 7. The excitation process in S_1_ and S_2_ by absorbing UV light may get hindered in the presence of the plasticizer, which is more prone to degradation in UV light [53]. Because of the negative outcome of the plasticizer incorporation on the phosphorescence intensity of the PP/S_1_ composites, they were not studied for the PP/S_2_ composites.

### 3.5. Mechanical Characteristics of PP/S_1_ and PP/S_2_ Composites

**Figure 8:**
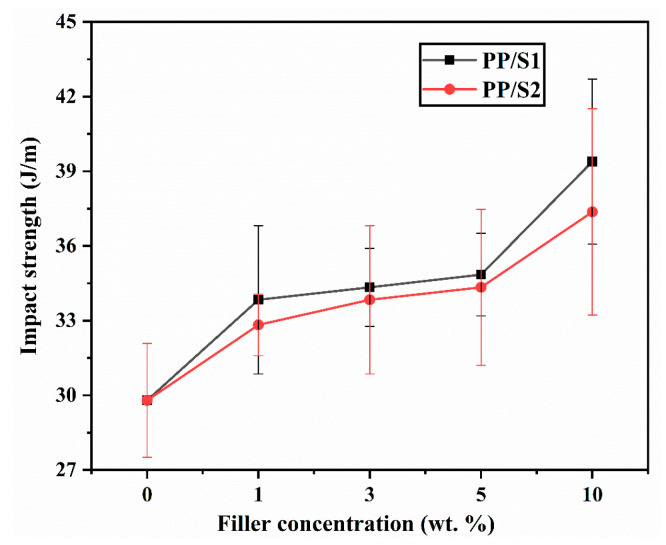
Impact strength of PP/S_1_ and PP/S_2_ composites.

For practical application purposes, the composites must exhibit suitable physical properties. Therefore, the Izod impact strength and tensile data of the PP with PP/S_1_ and PP/S_2_ composites were evaluated and are illustrated in Figure 8 and Figure 10, respectively.

**Figure 9:**
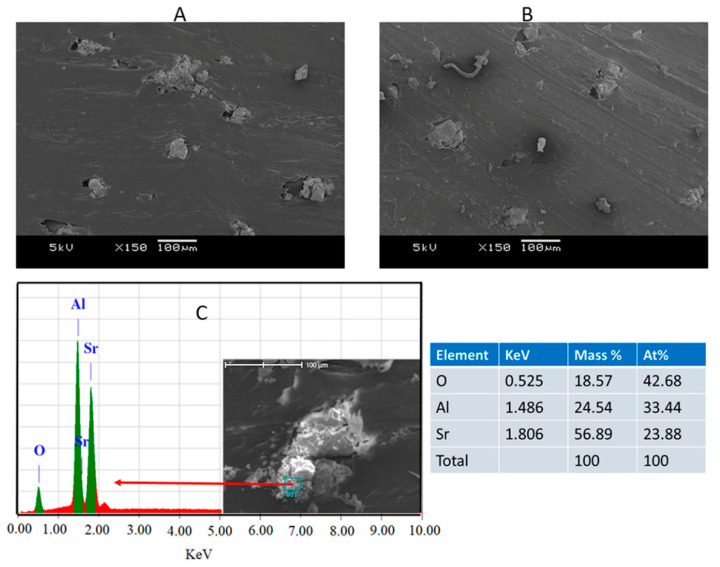
SEM images of PP/10S_1_ (**A**) and PP/10S_2_ (**B**) composites, and (**C**) SEM-EDS of PP/10S_1._

The impact strength of PP/S_1_ and PP/S_2_ composites increased gradually by increasing the S_1_ and S_2_ contents as shown in Figure 8. The PP composites with 10 wt.% filler (S_1_ and S_2_) showed the highest notch impact strength, which is ~ 32% greater than that for PP. The increase in the impact strength of the composites is because of the better interfacial adhesion among the PP and fillers (S_1_ and S_2_), which allows more efficient stress transfer. The distribution of fillers and the increased adhesion between the fillers and PP are visible in the SEM images of the composites with the highest S_1_ and S_2_ loading of 10 wt.% (Figure 9A,B).

**Figure 10:**
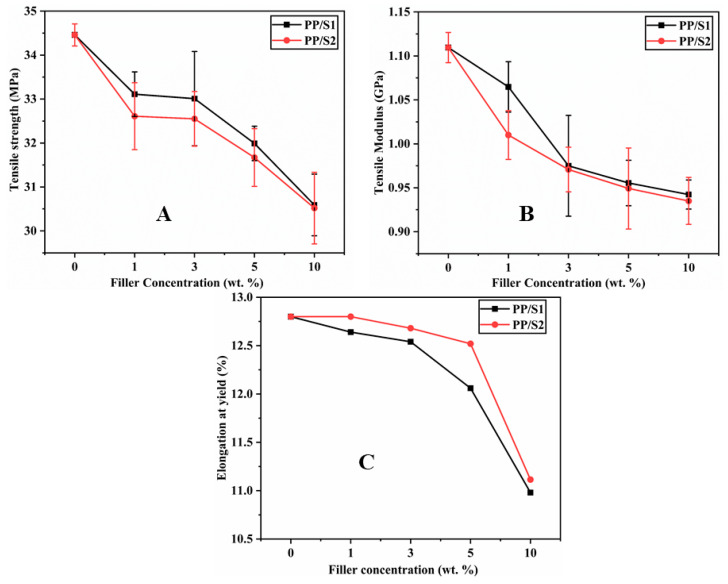
Tensile strength (**A**), tensile modulus (**B**), and elongation at yield (**C**) for PP/S_1_ and PP/S_2_ composites.

The tensile properties of PP and PP/S_1_ and PP/S_2_ composites are presented in Figure 10A,B, respectively. A gradual decrease in TS and tensile TM of the composites can be seen with the increase in the weight content of S_1_ and S_2_. In the highest filler loading (10 wt.% S_1_ and S_2_), the TS and TM decreased from 34.5 to 30.5 MPa and 1.1 to 0.94 GPa, respectively. The decrease in the tensile properties of PP/S_1_ and PP/S_2_ composites by increasing the filler loading can be due to the fact that the presence of inorganic fillers (S_1_ and S_2_) generally influences the elastic properties of PP because of their intrinsic stiffness and incapability to transfer the applied stress [37]. These observations are in agreement with the decrease in elongation at the yield values for these composites, as shown in Figure 10C. This is because of the decrease in PP ductility in the presence of S_1_ and S_2_ particles, which decreases the PP chain mobility. The inorganic-polymer composites often cause phase separation due to their incompatibility leading to a reduction in elongation at yield and break [54]. Additionally, the agglomeration of S_1_ and S_2_ filler, as shown in the SEM images (Figure 9A–C), adversely impacts the tensile modulus values.

## 4. Conclusions

In this study, strontium aluminate-based phosphors (S_1_ and S_2_) were melt-mixed with PP matrix to achieve the long afterglow properties. A long-lasting, PP encapsulated S_1_ and S_2_ composites with long afterglow properties, which lasts for hours, were obtained. The ATR-FTIR spectra confirmed that the melt-blending process was physical. Moreover, the luminescence spectra of the composite have a major excitation peak at 320 nm and an emission peak at 520 nm (S_1_; green) and 495 nm (S_2_; blue), respectively. The thermal studies show that the Tc and Tm of PP were not affected by the S_1_ and S_2_ fillers. However, there was a significant decrease in the crystallinity of the composites with S_2_ fillers, owing to the comparatively bulky chemical structure of these fillers. The impact strength of the resultant composites increased with the filler amount, but an adverse effect was witnessed on the TS and TM. These results demonstrated the satisfactory prospects for the formulation of phosphorescence films based on low-cost PP, which has great potential for applications in a new generation of light sources such as traffic signage and emergency signals.

## Figures and Tables

**Table 1:** DSC data of PP, PP/S_1_, and plasticized composites.

Material	*T_c_* (^°^C)	*T_m_* (^°^C)	Δ*H_m_* (J/g)	*X_c_* (%)
PP	121.9	164.6	87.0	42.0
PP/1S_1_	122.6	165.7	83.8	40.5
PP/3S_1_	122.7	164.5	83.2	40.2
PP/5S_1_	122.5	164.1	83.3	40.2
PP/10S_1_	122.9	164.2	83.8	40.5
PP/10S_1_/2.5P	122.0	164.0	82.5	39.9
PP10S_1_/5P	121.6	163.9	76.6	37.0

**Table 2:** DSC data of PP and PP/S_2_ composites.

Material	*T_c_* (^°^C)	*T_m_* (^°^C)	Δ*H_m_* (J/g)	*X_c_* (%)
PP	121.9	164.6	87.0	42.0
PP/1S_2_	120.9	163.5	74.9	36.2
PP/3S_2_	120.0	164.4	71.4	34.5
PP/5S_2_	120.1	164.6	68.6	33.1
PP/10S_2_	120.7	164.9	66.9	32.3

## Data Availability

The data presented in this study are available on request from the corresponding author.
